# Accuracy of stroke volume variation as a predictor of volume responsiveness in patients with raised intra-abdominal pressure

**DOI:** 10.1186/cc9488

**Published:** 2011-03-11

**Authors:** WO Bauer, M Cecconi, A Rhodes, W Bernal, J Wendon, G Auzinger

**Affiliations:** 1 King's College Hospital, London, UK; 2 St George's Hospital, London, UK

## Introduction

Dynamic predictors of fluid responsiveness such as stroke volume variation (SVV) are gaining popularity. Intra-abdominal hypertension (IAH) affects heart-lung interactions and may invalidate SVV as a preload indicator, as indeed suggested in a recent animal study [1]. We studied SVV in liver patients, who have a high incidence of raised intra-abdominal pressure (IAP).

## Methods

Patients admitted to a specialist liver ICU with acute or decompensated chronic liver disease were studied. All were in shock and received controlled mechanical ventilation. Cardiac output monitoring via transpulmonary thermodilution (PiCCO; Pulsion Medical Systems) and pulmonary artery catheterisation (CCombo; Edwards Lifesciences) was performed. Measurements before and after a 300 ml colloid bolus (Voluven; Fresenius Kabi) were recorded; fluid responsiveness was defined as an increase in stroke volume (SV) >10%. IAP was monitored via a Foley manometer and patients were divided into two groups: none/mild versus clinically significant IAH, cut-off value 15 mmHg. Volume responsiveness according to SVV and severity of IAH was analysed via receiver operating characteristic. Demographic parameters are displayed as the median and range.

## Results

Twenty-three measurements were made in 18 patients (in five patients, two fluid boluses were given on separate days). Median age was 45 years (47), 11 were females. Diagnoses were acetaminophen-induced acute liver failure (ALF, *n *= 6), acute decompensation of alcoholic liver disease (*n *= 4), Budd-Chiari syndrome (*n *= 3), seronegative ALF (*n *= 2), post-transplant septic shock (*n *= 2) and leptospirosis (*n *= 1). The median SOFA score was 18 (12), nor-epinephrine dose 0.26 μg/kg/minute (1.25). Clinically significant IAH was present in 15 measurements (IAP 17 to 27). Ten fluid boluses resulted in an increase in SV >10%. As a whole SVV failed to predict fluid responsiveness (area under curve (AUC) 0.53, *P *= 0.82). The subgroup with IAP <15 showed a trend towards significance (AUC 0.91, *P *= 0.06). In the latter group a SVV of 13.5% had 75% sensitivity and specificity in predicting fluid responders.

## Conclusions

SVV does not predict fluid responsiveness in patients with significant intra-abdominal hypertension. If IAP is mildly raised, higher cut-off levels for SVV may need to be considered.

## References

[B1] Renner J Crit Care Med 2009 37 650 658 10.1097/CCM.0b013e3181959864 19114894

